# Brain Functional and Structural Signatures in Parkinson’s Disease

**DOI:** 10.3389/fnagi.2020.00125

**Published:** 2020-05-26

**Authors:** Chunhua Liu, Jiehui Jiang, Hucheng Zhou, Huiwei Zhang, Min Wang, Juanjuan Jiang, Ping Wu, Jingjie Ge, Jian Wang, Yilong Ma, Chuantao Zuo

**Affiliations:** ^1^Shanghai Institute for Advanced Communication and Data Science, Shanghai University, Shanghai, China; ^2^Key Laboratory of Specialty Fiber Optics and Optical Access Networks, Joint International Research Laboratory of Specialty Fiber Optics and Advanced Communication, Shanghai University, Shanghai, China; ^3^PET Center, Huashan Hospital, Fudan University, Shanghai, China; ^4^Department of Neurology, Huashan Hospital, Fudan University, Shanghai, China; ^5^Center for Neurosciences, Feinstein Institute for Medical Research, North Shore-Long Island Jewish Health System, Manhasset, NY, United States; ^6^Institute of Functional and Molecular Medical Imaging, Fudan University, Shanghai, China; ^7^Human Phenome Institute, Fudan University, Shanghai, China

**Keywords:** Parkinson’s disease, brain network, pattern, ^18^F-FDG PET, MRI

## Abstract

The aim of this study is to explore functional and structural properties of abnormal brain networks associated with Parkinson’s disease (PD). ^18^F-Fluorodeoxyglucose positron emission tomography (^18^F-FDG PET) and T1-weighted magnetic resonance imaging from 20 patients with moderate-stage PD and 20 age-matched healthy controls were acquired to identify disease-related patterns in functional and structural networks. Dual-modal images from another prospective subject of 15 PD patients were used as the validation group. Scaled Subprofile Modeling based on principal component analysis method was applied to determine disease-related patterns in both modalities, and brain connectome analysis based on graph theory was applied to verify these patterns. The results showed that the expressions of the metabolic and structural patterns in PD patients were significantly higher than healthy controls (PD1-HC, *p* = 0.0039, *p* = 0.0058; PD2-HC, *p* < 0.001, *p* = 0.044). The metabolic pattern was characterized by relative increased metabolic activity in pallidothalamic, pons, putamen, and cerebellum, associated with metabolic decreased in parietal–occipital areas. The structural pattern was characterized by relative decreased gray matter (GM) volume in pons, transverse temporal gyrus, left cuneus, right superior occipital gyrus, and right superior parietal lobule, associated with preservation in GM volume in pallidum and putamen. In addition, both patterns were verified in the connectome analysis. The findings suggest that significant overlaps between metabolic and structural patterns provide new evidence for elucidating the neuropathological mechanisms of PD.

## Introduction

Parkinson’s disease (PD) is a complex, chronic, and neurodegenerative disorder, pathologically characterized predominately by a loss of substantia nigra pars compacta dopaminergic neurons, manifesting in functional and structural alterations throughout the brain (Lee and Trojanowski, 2006; Choi et al., 2013; Rocha et al., 2018). Pathological studies have shown that the cortical and subcortical regions are widely involved in PD pathology (Braak et al., 2003). Current efficient diagnostic tools for PD neuroimaging—including magnetic resonance imaging (MRI), positron emission tomography (PET), and single-photon emission computed tomography (SPECT)—rely on different principles that could be useful depending on the research or clinical setting available (Politis, 2014). In particular, ^18^F-fluorodeoxyglucose positron emission tomography (^18^F-FDG PET) has been used to localize and quantify abnormal brain energy metabolism *in vivo* and recently been used in metabolic connectivity studies to identify different disease-specific patterns (Titov et al., 2017). It is increasingly used in routine clinical practice (Teune et al., 2013). For instance, spatial covariance analysis of ^18^F-FDG PET data consistently reveals the presence of a stereotyped spatial covariance pattern associated with different stages of motor symptoms in PD patients. The metabolic Parkinson’s disease-related pattern (PDRP) associated with motor symptoms is characterized by increased metabolism in the putamen, globus pallidus, bilateral thalamus, and pontine, and relatively decreased metabolism in the premotor and parieto-occipital cortex (Ma et al., 2007; Eidelberg, 2009; Spetsieris and Eidelberg, 2011; Spetsieris et al., 2013; Wu et al., 2013; Ko et al., 2017; Tomše et al., 2017). The reproducibility of metabolic PDRP in different cohorts, according to the extensive literature, indicated that the metabolic PDRP was a reliable marker of disease across various ethnic groups and PET instrumentations as well as imaging protocols (Schindlbeck and Eidelberg, 2018). Moreover, the PDRP expression values (subject scores) from various studies have also shown significant positive correlations with disease progression and have decreased after effective treatments of motor symptoms (Huang et al., 2007; Peng et al., 2014b).

While metabolic PDRP can effectively reveal the abnormal metabolic function in PD, these functional abnormalities may also have corresponding changes in neuroanatomical structures. MRI, as a noninvasive examination and cost-effective imaging technique, has become more and more important to the study of neurodegenerative diseases, which revealed the structural and functional alterations underlying these conditions. For example, T1-weighted structural MRI is able to measure the volume/thickness alterations of the gray and white matters in the subcortical and cortical areas associated with PD (Wilson et al., 2019). Currently, imaging studies have provided preliminary evidence for structural abnormalities in the brain of PD patients, especially brain atrophy. Given that prolonged metabolic derangement causes atrophy, the spatial pattern of atrophy demonstrated an overlap with the metabolic PDRP topography, as well as with intrinsic networks present in healthy brain (Zeighami et al., 2015). However, it is still under speculation whether both spatial patterns of metabolic function and atrophy are effective to address PD progression at the same time.

In response to this issue, we explored the topographical relationship between both spatial patterns of metabolic function and atrophy. In addition, we also explored corresponding disease intrinsic networks defined using brain connectome analysis based on graph theory. This analysis method is an innovative approach that has revealed fundamental aspects of brain structural or functional network organization, and it can reveal abnormalities in network characteristics associated with neurological diseases and the potential impact of the disease network on brain information processing (Fox, 2018). Currently, graph theory in conjunction with spatial covariance analysis was used to examine the topology of disease networks in metabolic PDRP (Ko et al., 2017). The results in that study showed that disease networks defined by the spatial covariance analysis in PD patients exhibit exaggerated small world property, suggesting that it is more beneficial to elucidate the pathological basis of PD from the perspective of PD-related metabolic functional alterations in disease intrinsic networks. Nevertheless, it remains to be determined whether such alterations also occur in PD-related structural networks.

The primary objective of this work was to characterize inherently metabolic PDRP and structural PDRP together in PD patients and explore their correlations. A secondary aim was to explore whether the disease brain networks derived from metabolic or structural PDRP from graph theory has abnormal topological characteristics. We also hypothesized that there are abnormal topological characteristics in the disease structure and metabolic networks in the PD.

## Materials and Methods

**Figure 1:**
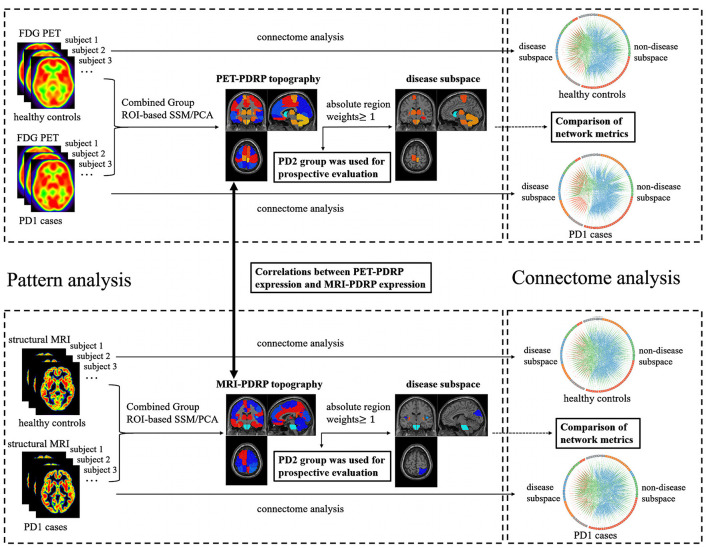
The overall framework of the experimental process used in this study.

Figure 1 provides the general framework of our study. First, both ^18^F-FDG PET and T1-weighted structural MRI scans from PD1 and HC subjects in cohort A were analyzed using spatial covariance analysis to identify a significant region-of-interest (ROI)-based metabolic PDRP (PET-PDRP) topography and a structural PDRP (MRI-PDRP) topography. Both topographies and corresponding pattern expression values were compared with each other. Data from PD2 subjects in cohort B were used for a prospective evaluation of both metabolic and structural PDRPs in single cases. Second, based on the customized criteria, we identified some salient abnormal brain regions from the PET-PDRP or MRI-PDRP topographies as network nodes in the disease subspace, and the remaining regions constituted the nondisease subspace. Finally, to explore network properties in the disease and nondisease subspaces in PD patients, connectome analysis based on graph theory was performed from each group. We calculated the corresponding network metrics between the disease and nondisease subspace and compared differences in network metrics for the PD groups relative to the corresponding control values.

### Subjects

This study included two different cohorts (cohort A and B) of healthy subjects and PD patients (Table 1). Cohort A included 20 nondemented patients with PD and 20 age-matched healthy controls recruited from Huashan Hospital (Shanghai, China) in one project conducted from January 2014 to September 2015. Cohort B included 15 nondemented patients with PD recruited from Huashan Hospital (Shanghai, China) in another project conducted from January 2013 to September 2013. All subjects in cohorts A and B underwent both ^18^F-FDG PET and T1-weighted structural MRI scans. All patients were scanned and clinically examined by two senior neurologists specializing in dyskinesia before their inclusion in the study. The PD diagnosis was according to the United Kingdom Brain Bank criteria (Hughes et al., 1992). Patients with PD have no dementia, supranuclear gaze abnormalities, or ataxia, and are not caused by encephalitis or antipsychotic medication. The Unified Parkinson’s Disease Rating Scale (UPDRS) motor examination was administered at least 12 h after the cessation of antiparkinsonian medications. Mini-mental State Examination (MMSE) was administered synchronously.

**Table 1:** Demographic characteristics of PD patients and healthy controls.

	Cohort A		Cohort B	*p* value	
	HC	PD1	PD2		
*N*	20	20	15	PD1-HC	PD2-HC
Age (years)	59.6 ± 7.2	62.4 ± 7.3	62.8 ± 4.9	0.230^a^	0.148^a^
Gender (F/M)	12/8	10/10	3/12	0.525^b^	**0.018^b^**
UPDRS	-	21.6 ± 11.2	21.6 ± 10.5	-	-
Speech	-	0.5 ± 0.5	0.5 ± 0.5	-	-
Facial expression	-	1.2 ± 1.0	1.3 ± 1.0	-	-
Static tremor	-	2.2 ± 2.1	2.1 ± 2.3	-	-
Postural tremor	-	1.2 ± 1.1	1.1 ± 1.0	-	-
Rigidity	-	4.6 ± 3.6	4.1 ± 5.0	-	-
Gait	-	0.8 ± 0.6	0.8 ± 0.6	-	-
MMSE	-	28.8 ± 1.7	27.8 ± 1.9	-	-

*Age and clinical rating are given as mean ± standard deviation. UPDRS, Unified Parkinson’s Disease Rating Scale. ^a^*p* value was obtained by the two-sample two-tailed *t*-test between healthy controls and PD patients. ^b^*p* value was obtained by the χ^2^ test between healthy controls and PD patients. Bold value indicated a significant difference between healthy controls and PD patients*.

All healthy controls received the same clinical scanning procedures as PD patients. Exclusion criteria for all subjects included: (a) primary psychiatric illness; (b) central nervous system comorbidities; and (c) abnormal neurological examination.

Ethical permission for the study was obtained from the Research Ethics Committee of Huashan Hospital, Shanghai, China (approval number: KY2013-336). Written informed consent was obtained from each subject after providing detailed instructions of the procedures.

### Acquisition Protocol

#### ^18^F-FDG PET

All subjects underwent ^18^F-FDG PET examination at resting state. PET scans were performed with a Siemens Biograph 64 HD PET/CT (Siemens, Germany) in three-dimensional (3D) mode. All subjects were fasted for at least 6 h before scanning. After intravenous injection of 185 MBq ^18^F-FDG, the PET scan was started after a 45-min rest in a quiet and dimly lit environment. Prior to the PET scan, a low-dose CT transmission scan was performed for attenuation correction. The PET scan was performed in 3D mode for 10 min. All PET data were reconstructed using a 3D ordered subset expectation maximization algorithm and corrected for random coupling, scattering, and radioactive decay.

#### T1-Weighted Structural MRI

All MRI measurements were obtained on a 3-T GE Discovery MR750 Scanner (Milwaukee, WI, USA) equipped with a circular polarized eight-channel head matrix coil at the Department of Radiology of Huashan Hospital of Fudan University, China. High-resolution, T1-weighted, 3D anatomical brain images were obtained using an inversion recovery prepared fast spoiled gradient recalled sequence (repetition time = 11.1 ms; echo time = 5.0 ms; flip angle = 20°; matrix resolution = 256 × 256; voxel size = 1 × 1 × 1 mm^3^; field of view = 240 mm^2^; slice thickness = 1.0 mm; 146 slices without slice gap, transverse acquisition), with the scan range from the calvarium to foramen magnum.

### Data Preprocessing

Data preprocessing for both PET and MRI images was done using Statistical Parametric Mapping 12 (the Wellcome Department of Neurology, London, UK) package implemented in Matlab2016b (Mathworks Inc.). First, ^18^F-FDG PET scan for each subject was aligned with corresponding T1-weighted MRI scan. Second, MRI images were segmented into gray matter (GM), white matter (WM), and cerebrospinal fluid (CSF) tissue probability maps. Then, the GM map was registered to the Montreal Neurological Institute (MNI) stereotaxic template using nonlinear transformation parameters. The aligned PET image was also normalized to the MNI template using the same transformation parameters. Finally, the normalized MRI and PET images were smoothed equivalent to a convolution with an isotropic Gaussian kernel of 8 mm to increase signal-to-noise ratios.

### Pattern Analysis

Pattern analysis was performed using ScanVP 7.0w package implemented in Matlab2016b^1^ (Eidelberg, 2009; Spetsieris and Eidelberg, 2011). The PD-related covariance pattern was generated using ROI-based spatial covariance mapping algorithms known as Scaled Subprofile Modeling based on principal component analysis (SSM/PCA). In this step, the smoothed ^18^F-FDG PET data from the combined PD1 patients and healthy subjects in cohort A was used as inputs. First, we performed a logarithmic transformation on the mean glucose metabolism within each region for each subject, and the subject × ROI data matrix was obtained. Second, the PCA was executed on the matrix to identify a disease-related spatial covariance pattern reflected major sources of variation. This pattern was a linear combination of selected principal components (PCs), so that the expression values corresponding to the pattern could maximally separate the PD patients from the control subjects. The number of PCs was determined by the lowest Akaike information standard (AIC) value in the logistic regression model. Third, the regional weights of the pattern were *z*-scored based upon the mean and standard deviation of all regions (Spetsieris et al., 2013). Therefore, the subject expression of this pattern in a prospective subject can be computed using an ROI-based topographic profile rating (TPR) algorithms (Eidelberg, 2009; Spetsieris et al., 2013). Finally, the resulting expression was *Z*-transformed using the subject expressions of the control subjects participating in the pattern identification. In this study, we preselected 95 ROIs. In addition to 90 ROIs from the automated anatomical labeling (AAL) atlas (Tzourio-Mazoyer et al., 2002), we also included five other regions where functional imaging studies in PD commonly report altered metabolism, including bilateral cerebellum, bilateral pons, and cerebellar vermis. The MRI images were analyzed by the same process as above.

As a result, a significant ROI-based metabolic PDRP (and structural) topography was identified from ^18^F-FDG PET (and T1-weighted GM MRI) scans of combined PD1 and HC subjects in cohort A. Subject expressions of corresponding PDRP were then computed for all scans in the combined PD1 and HC subjects and *Z*-scored using subject expressions of the HC. We validated corresponding PDRP topography by computing its expression in the ^18^F-FDG PET (and T1-weighted GM MRI) scans from PD2 subjects in cohort B. The corresponding PDRP expressions of PD2 subjects were *Z*-scored the same way as above and then compared with those of the original subjects in PD1 and HC. The diagnostic power of the corresponding PDRP expressions for discriminating PD patients from healthy controls was evaluated by the area under the curve (AUC) in the receiver operating characteristic (ROC) curves. ROC analysis in cohort B was obtained by comparing PD2 and HC groups. ROC analysis in cohorts A and B (cohort A + B) was obtained by comparing combined PD1 and PD2 groups compared to HC group. We also evaluated whether the combination of PET-PDRP and MRI-PDRP expressions could improve diagnostic power. In addition, correlation analysis of corresponding PDRP expression and clinical ratings in PD patients in cohorts A and B were also performed.

### Correlation Analysis Between PET-PDRP and MRI-PDRP Topographies

The metabolic and structural topographies were assessed by ROI-based correlation of regional weights in a set of salient abnormal brain regions. The correlation between the two patterns was calculated using only the regions with absolute values ≥1.0 (Ge et al., 2018). The pattern expression for PET-PDRP was also correlated with corresponding MRI-PDRP expression in combined PD1 (or PD2) and HC samples.

### Brain Connectome Analysis

Graph theory was used to explore network properties in brain connectome analysis. Globally normalized glucose metabolism within each ROI was used to construct a region × region correlation matrix across each of three individual groups (PD1, PD2, and HC groups). In each correlation matrix, functional connectivity (FC) between each pair of regions was calculated by partial correlation coefficient between ROI values for local FDG uptake in each pair of regions, across participants, to conduct for the control of age and gender effects (Lo et al., 2010; Duan et al., 2017; Jiang et al., 2017). We used a sparsity (or named cost, representing present connections as a percentage of all possible connections) threshold to generate a series of undirected graphs (Baggio et al., 2014; Duan et al., 2017). The correlation matrices were thresholded at a range of sparsity thresholds, in 0.01 steps (sparsity_min_: 0.01: sparsity_max_). The minimum sparsity guaranteed that networks of all groups (HC, PD1, and PD2) were fully connected, and no nodes were fragmented. The maximum sparsity selected 0.5 because the randomness of the network larger than this threshold would increase, and the results would be unreliable (Hosseini et al., 2012). At each threshold, we calculated the following network metrics: (1) the clustering coefficient (*C*, quantification of the degree to which nodes in a graph tend to cluster together and a representation of network segregation, measuring the local information transmission capability in a network); (2) the characteristic path length (*L*, the average number of connections on the shortest path between any two regions in a network and a marker of network integration, measuring the global information transmission capability in a network); and (3) small worldness (*S*, the balance between local segregation and global integration).

Next, we divided the brain into disease subspace and nondisease subspace based upon the PET-PDRP topographies for further analysis. The nodes in the disease subspace consisted of the salient abnormal brain regions (absolute regional weight ≥1.0; high local contributions to overall PDRP activity) identified by the PDRP topography and the remaining regions constituting the nondisease subspace (absolute regional weight <1.0; low local contributions to overall PDRP activity). We calculated the corresponding network metrics among disease subspace, nondisease subspace, and whole brain, and we compared differences in network metrics for the PD1/PD2 groups relative to HC control values.

The graph theoretical analyses of brain structural network were the same as in the procedure for metabolic brain network but based on T1-weighted GM imaging data. It was worth noting that disease subspace and nondisease subspace in brain structural network were divided according to MRI-PDRP topography.

Brain connectome analysis was performed using Brain Connectivity Toolbox^2^ and Graph Analysis Toolbox (Hosseini et al., 2012) implemented in Matlab2016b.

### Statistical Analysis

Differences in PET-PDRP (and MRI-PDRP) expressions between patients and healthy controls were evaluated using two-sample *t*-tests. PDRP expressions of patients were correlated with corresponding MMSE, UPDRS, and six motor items (speech, facial expression, static tremor, postural tremor, rigidity, and gait) by computing Pearson’s correlations. Regional weight and pattern expression between the two PDRPs identified in the ^18^F-FDG PET scans and structural MRI scans was compared using Pearson’s correlations. In order to determine the significance of the differences in network metrics between the patient group and HC group in each subspace, a permutation test repeated 1,000 times was used (Hosseini et al., 2012; Ko et al., 2017). All statistical tests were performed using Matlab2016b (*p* < 0.05, two-tailed).

## Results

### Abnormal Disease Topographies in Pattern Analysis

#### PET-PDRP Identification and Validation

**Figure 2:**
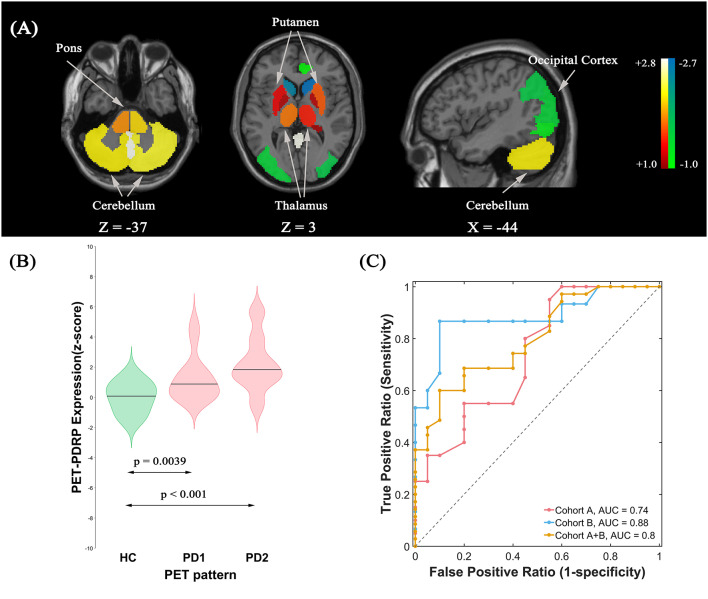
**(A)** Metabolic Parkinson’s disease-related pattern (PET-PDRP) identified by Scaled Subprofile Modeling (SSM) multivariate analysis of ^18^F-FDG PET scans from 20 (PD) patients in PD1 and 20 age- and gender-matched healthy controls. The pattern was characterized by relative increased metabolic activity in the pallidothalamic, pons, bilateral putamen, and cerebellum, associated with metabolic decreased in parietal–occipital areas. **(B)** Subject expressions for the PET-PDRP topography measured using violin plots in the HC, PD1, and PD2 scans (horizontal lines indicate group medians). Significant increases in pattern expression were seen in PD1 original derivation subjects and PD2 validation subjects with respect to HC control values. **(C)** Receiver operating characteristic (ROC) curve for discriminating PD patients from healthy controls.

The ROI-based SSM/PCA multivariate analysis of ^18^F-FDG PET data from cohort A investigated the first four PCs that explained 55.4% of the subject × ROI variance. The PET-PDRP topography generated from a linear combination of PC1, PC3, and PC4 with expression successfully discriminated PD1 patients and healthy controls, and produced the lowest AIC value in the logistic regression model. The pattern was characterized by relative increased metabolic activity in pallidothalamic, pons, bilateral putamen, and cerebellum, associated with metabolic decrease in parietal–occipital areas (Figure 2A). Subject expressions for the PET-PDRP topography were significantly elevated (*p* = 0.0039) in PD1 compared to HC subjects (Figure 2B). Significant increases in pattern expression were also seen in PD2 validation subjects with respect to HC control values (*p* < 0.001). Subject PET-PDRP expressions were without a difference between PD1 and PD2 subjects (*p* = 0.097). ROC analysis revealed an AUC = 0.74 (95% confidence intervals of 0.59–0.89; Figure 2C) to distinguish the PD patients from the controls in cohort A. The ROC curves also showed that PET-PDRP expression accurately distinguished PD patients from the control individuals in cohorts B and A + B. The AUC values were 0.88 (95% confidence intervals of 0.75–1.00) and 0.80 (95% confidence intervals of 0.68–0.92) for subjects in cohorts B and A + B, respectively (Figure 2C).

#### MRI-PDRP Identification and Validation

**Figure 3:**
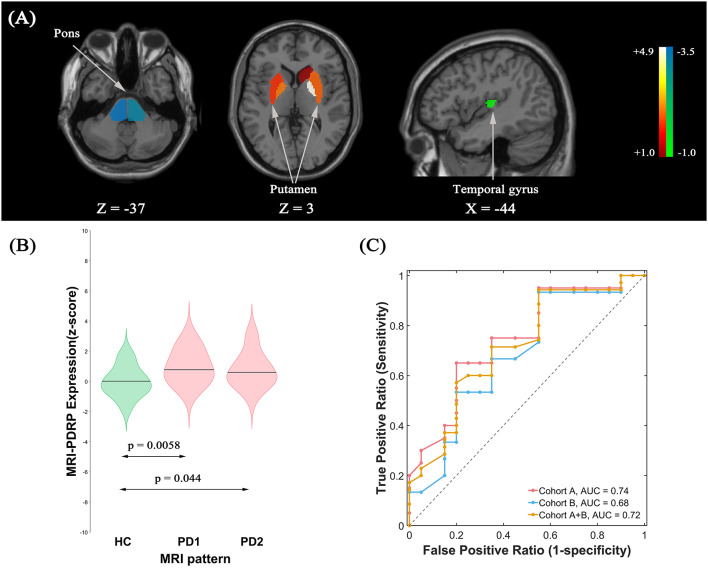
**(A)** Structural Parkinson’s Disease-Related Pattern (MRI-PDRP) derived from the same subjects. The pattern was characterized by relative decreased gray matter (GM) volume in bilateral pons, bilateral transverse temporal gyrus, left cuneus, right superior occipital gyrus, and right superior parietal lobule, associated with preservation in GM volume in bilateral pallidum and bilateral putamen. **(B)** Subject expressions for the MRI-PDRP topography measured using violin plots in the HC, PD1 and PD2 scans (horizontal lines indicate group medians). Significant increases in pattern expression were seen in PD1 original derivation subjects and PD2 validation subjects with respect to HC control values. **(C)** ROC curve for discriminating PD patients from healthy controls.

The pattern analysis of MRI images examined the first four PCs accounting for 52.4% subject × ROI variance. An MRI-PDRP was generated by a linear combination of PC2 and PC3. The pattern was characterized by relative decreased GM volumes in bilateral pons, bilateral transverse temporal gyrus, left cuneus, right superior occipital gyrus, and right superior parietal lobule, associated with preservation in GM volumes in bilateral pallidum and bilateral putamen (Figure 3A). Subject expressions for the MRI-PDRP topography were significantly elevated (*p* = 0.0058) in PD1 compared to HC subjects (Figure 3B). Significant increases in pattern expression were seen in PD2 validation subjects with respect to HC control values (*p* = 0.044). Subject MRI-PDRP expressions were not different between PD1 and PD2 subjects (*p* = 0.599). As for ROC curves distinguishing the PD patients from the normal controls, AUC values were 0.74 (95% confidence intervals of 0.59–0.90), 0.68 (95% confidence intervals of 0.50–0.86), and 0.72 (95% confidence intervals of 0.57–0.86) for subjects in cohorts A, B, and A + B, respectively (Figure 3C).

### Correlation Analysis

#### Correlations Between PDRP and Clinical Scales

**Figure 4:**
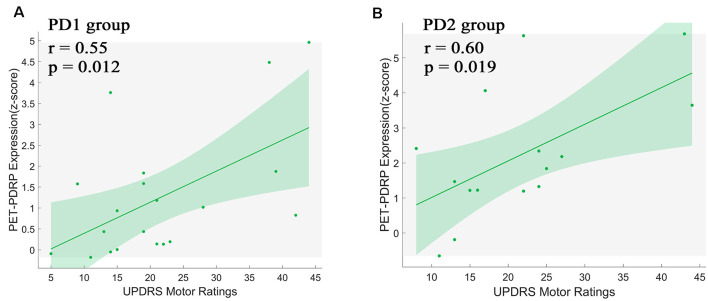
PET-PDRP expressions in individual patients correlated with UPDRS motor ratings in the **(A)** original derivation subjects as well as in the **(B)** subsequent validation subjects. Shaded areas represent 95% confidence of intervals. UPDRS, Unified Parkinson’s Disease Rating Scale.

**Figure 5:**
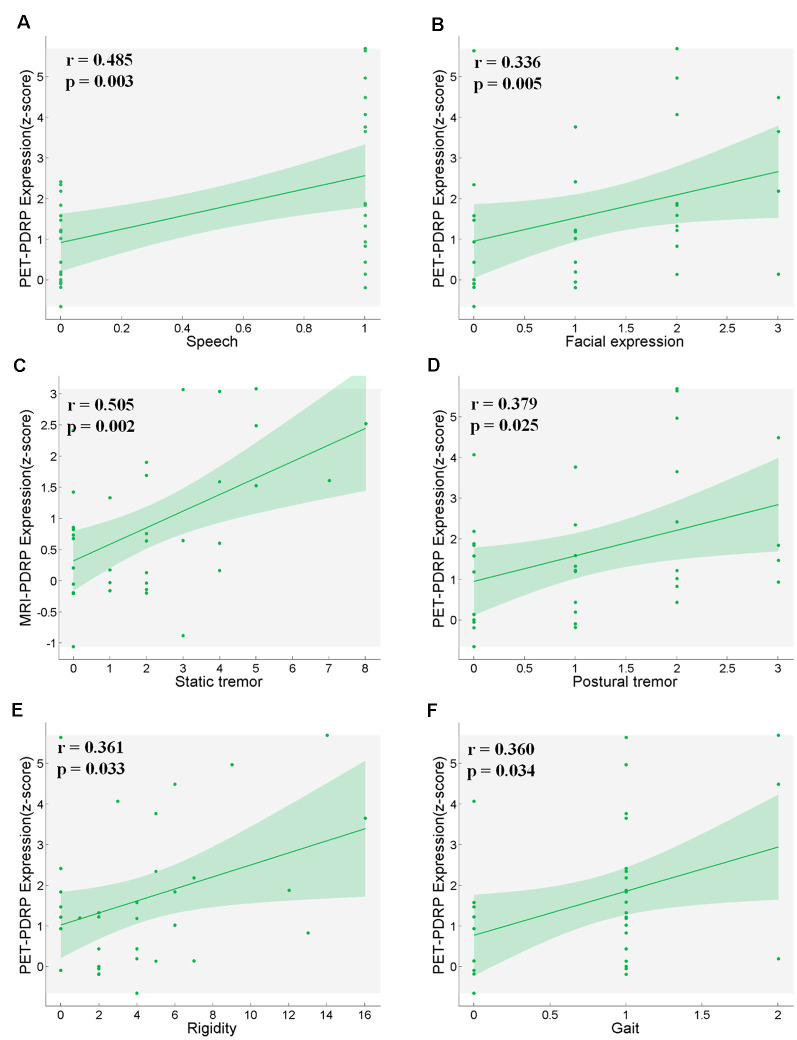
PET-PDRP expressions in PD patients correlated with their levels of **(A)** speech, **(B)** facial expression, **(D)** postural tremor, **(E)** rigidity and **(F)** gait. MRI-PDRP expressions in PD patients correlated with the levels of **(C)** static tremor. Shaded areas represent 95% confidence of intervals.

PET-PDRP and MRI-PDRP expressions were not correlated with MMSE in the PD group. The correlations between PET-PDRP expressions and the corresponding UPDRS within each PD group are shown in Figure 4. In PD1 group, PET-PDRP expressions in patients correlated positively with UPDRS motor ratings (*r* = 0.55, *P* = 0.01; Figure 4A). In PD2 group, PET-PDRP expressions in patients also correlated with UPDRS motor ratings (*r* = 0.60, *P* = 0.02; Figure 4B). In a combined PD group from PD1 and PD2, PET-PDRP expressions had positive correlations with UPDRS motor ratings (*r* = 0.54, *P* < 0.001). MRI-PDRP expressions were also associated with UPDRS motor ratings in PD1 group (*r* = 0.51, *P* = 0.02) but not PD2 group. In a combined PD group (PD1 + PD2), MRI-PDRP expressions were not correlated with UPDRS motor ratings. The correlation results between PET-PDRP expressions, MRI-PDRP expressions, and the six motor items of UDRPS are shown in Figure 5. PET-PDRP expressions in patients correlated positively with the scores of speech (*r* = 0.485, *P* = 0.003), facial expression (*r* = 0.336, *P* = 0.0049), postural tremor (*r* = 0.379, *P* = 0.025), rigidity (*r* = 0.361, *P* = 0.033), and gait (*r* = 0.360, *P* = 0.034). MRI-PDRP expressions in patients correlated positively with the scores of static tremor (*r* = 0.505, *P* = 0.002).

#### Correlations Between PET-PDRP and MRI-PDRP

**Figure 6:**
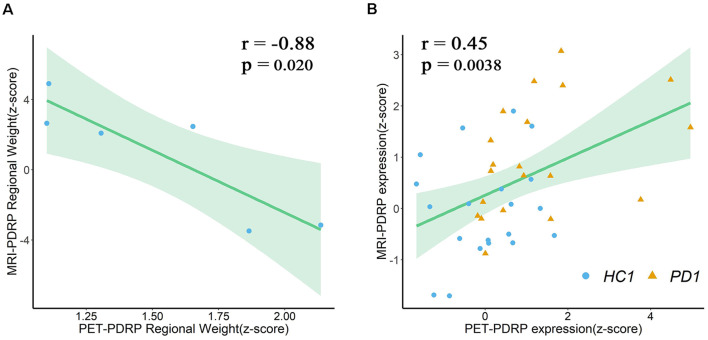
**(A)** Regional weights on PET-PDRP and MRI-PDRP in a set of salient abnormal brain regions were correlated. **(B)** PET-PDRP expressions in combination with PD1 patients and healthy controls and MRI-PDRP expressions in the same subjects were correlated. Shaded areas represent 95% confidence of intervals.

In the two PDRPs, six regions were salient abnormal brain regions (absolute region weight ≥1.0) in both patterns, including bilateral pons, bilateral pallidum, and bilateral putamen (Supplementary Tables S1, S2). Regional weights between the two PDRPs of these regions were negatively correlated (*r* = −0.88, *P* = 0.02; Figure 6A). Two pattern expression of the combined PD1 patients and healthy controls showed positive correlations (*r* = 0.45, *P* = 0.0038; Figure 6B) but not associated with combined PD2 and HC subjects or combined PD1, PD2, and HC subjects. The correlations between two pattern expressions in the single PD1 group was found (*r* = 0.42, *P* = 0.064) but not found in single PD2 group or combined PD1 and PD2 subjects.

### Brain Connectome Analysis

#### PD-Related Metabolic Network

**Figure 7:**
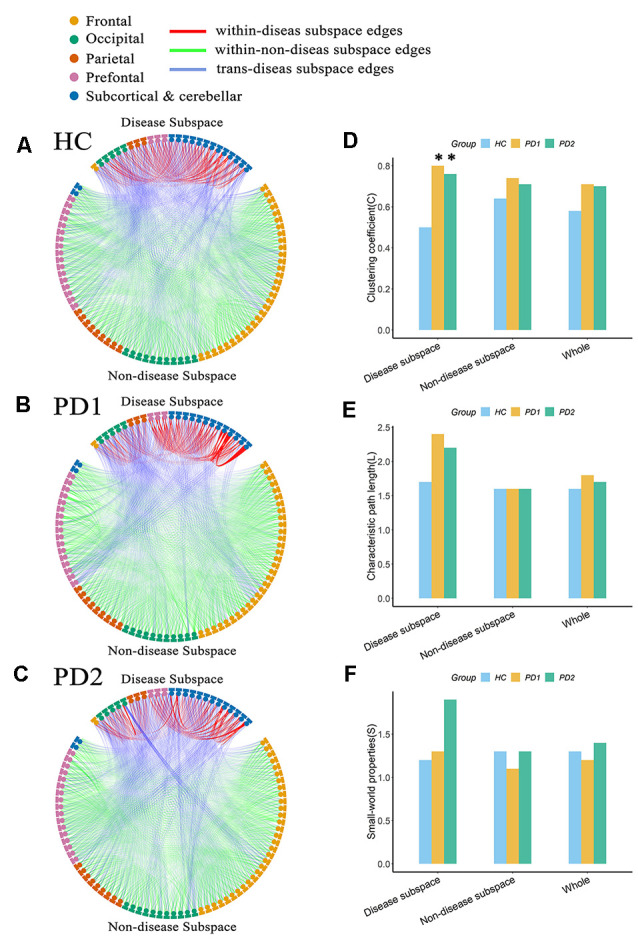
Node-to-node functional connectivity (FC) in the **(A)** HC, **(B)** PD1, and **(C)** PD2 groups in the disease subspace and nondisease subspace, and **(D–F)** network attributes. **(A)** Binary functional network in the HC group at sparsity 38%. **(B)** Binary functional network in the PD1 group at sparsity 38%. **(C)** Binary functional network in the PD2 group at sparsity 38%. **(D)** Comparison of the clustering coefficient among HC, PD1, and PD2 groups in different spaces at sparsity 38%. **(E)** Characteristic path length. **(F)** Small world coefficient. In **(A–C)**, the nodes in the upper part of the circle represent brain regions in the disease subspace (25 ROIs), and the nodes in the lower part of circle represent brain regions in the nondisease subspace (70 ROIs). Colors of nodes on the circle represent different anatomical classifications of corresponding brain regions based on existing literature (Wang et al., 2007; Bai et al., 2011). The lines within the circle represent FC between pairs of nodes, where the red lines (within-disease subspace edges) indicate FC between nodes within the disease subspace; the green lines (within-nondisease subspace edges) indicate FC between nodes within the nondisease subspace; the blue lines (trans-disease subspace edges) indicate FC between the disease subspace nodes and the nondisease subspace nodes. The asterisk refers to significant differences between the PD compared to HC groups (*p* < 0.05).

Connectome analysis was used to reveal abnormalities in network features associated with PD. In our study, in order to determine whether this feature is different between PD patients and healthy subjects, we separated each group’s network into two discrete subspaces. Eventually, 25 nodes constituted the disease subspace, which had a higher local contributions to overall PET-PDRP activity (for more details, please refer to Supplementary Table S1). The remaining 70 brain regions served as nodes in the nondisease subspace. The sparsity_min_ (ensuring both the disease subspace and nondisease subspace are fully connected) of the HC, PD1, and PD2 groups was 15, 38, and 28%, respectively. Network metrics in each group were computed with a sparsity threshold ranging from 38% to 50%. At sparsity 38%, the clustering coefficient for the disease subspace in the PD1 group was significantly (*P* < 0.05) increased compared with the HC group (PD1, 0.80; HC, 0.50; Figures 7A,B,D). The characteristic path length had a tendency to increase in PD1 compared to HC (PD1, 2.42; HC, 1.67; Figures 7A,B,E). However, significant differences were not observed for the small-worldness coefficient. Indeed, permutation analysis showed that, in the entire sparsity threshold range (38–50%), the clustering coefficient of PD1 group in the disease space increased significantly (*P* < 0.05); in the sparsity threshold range of 41–50%, the characteristic path length also increased significantly (*P* < 0.05; Supplementary Figure S1).

The clustering coefficient for the disease subspace was significantly (*P* < 0.05) increased in PD2 compared to HC over the sparsity range of 38–42% (for example, at sparsity 38%, C in PD2 is 0.76; C in HC is 0.50; Figures 7A,C,D). The small world coefficient for the disease subspace in PD2 group increased significantly (*P* < 0.05) at sparsity range of 39–41% (Supplementary Figure S2) and had an increasing trend at sparsity 38% (S in PD2, 1.91; S in HC, 1.19; Figures 7A,C,F). The characteristic path length (had a tendency to increase, such as, at sparsity 38%, L in PD2 is 2.16; L in HC is 1.67; Figures 7A,C,E) did not show group differences (Supplementary Figure S2). Group differences of network metrics were not significant for the nondisease subspace or for the whole brain.

#### PD-Related Brain Structural Network

According to the local contributions to overall MRI-PDRP activity, 11 nodes constituted the disease subspace (Supplementary Table S2), and the remaining 84 brain regions served as nodes in the nondisease subspace. PD-related brain structural network was also analyzed based on T1-weighted GM imaging data in different spaces. In the HC group, the minimum sparsity in which all nodes became fully connected in both the disease subspace and nondisease subspace was 20%, PD1 group was 32%, and PD2 group was 17%. Network metrics were computed at a sparsity range of 32–50% for the comparisons between PD1 (or PD2) and HC. At sparsity 32%, increased path length and slightly elevated small-world coefficients for the disease subspace were observed in PD1 group but not PD2 group (L in PD1, 2.15; L in PD2, 1.70; L in HC, 1.42; S in PD1, 2.88; S in PD2, 1.05; S in HC, 1.28; Supplementary Figure S3). As above, in the nonparametric permutation test analysis, we also found that increased path length (in the sparsity range of 38–47% and 49–50%) and slightly elevated small-world coefficient (at sparsity 39% and 50%) over corresponding sparsity range for the disease subspace in PD1 group but not PD2 group (Supplementary Figures S4, S5). Group differences of network metrics were not significant for the nondisease subspace or for the whole brain.

## Discussion

In this study, we investigated ^18^F-FDG PET-based metabolic covariance pattern (PET-PDRP) and T1 MRI-based structural covariance pattern (MRI-PDRP) associated with PD for the same patients. The MRI-PDRP topography revealed brain region-level abnormalities containing a large number of cortical neurons that partially overlap with the metabolic pattern derived from ^18^F-FDG PET scans. Connectome analysis also showed that the topological organization for the disease network in the PET-PDRP and MRI-PDRP topographies were significantly disrupted. These findings provide new evidence for elucidating the neuropathological mechanisms of PD.

### Reproducible Metabolic PDRP Topography

Using spatial covariance analysis, we reproduced a metabolic PDRP topography (PET-PDRP) that was compatible with previous imaging studies in both American and Chinese PD patients (Ma et al., 2007; Wu et al., 2013; Ko et al., 2017; Schindlbeck and Eidelberg, 2018). In our study, the PET-PDRP expression in PD patients was significantly elevated in the original derivation subjects and the subsequent validation subjects. The regional metabolic dysfunction within this abnormal topography could describe abnormal cerebral metabolism or blood flow and reveal clinical disability and treatment response in patients with PD (Hirano et al., 2008; Eidelberg, 2009; Wu et al., 2013; Ko et al., 2017). This topography revealed the presence of abnormal metabolic changes at key nodes of the cortico-striato-pallido-thalamo-cortical (CSPTC) loops and other related anatomical/functional pathways, which is in line with previous reports (Eidelberg, 2009; Poston and Eidelberg, 2009; Tang and Eidelberg, 2010). The abnormalities in the CSPTC circuits are commonly associated with the clinical manifestations of akinetic rigid in PD patients, but they do not fully explain other disease manifestations, such as tremors (Wichmann and Delong, 2007; Zaidel et al., 2009; Wu et al., 2013). By contrast, relative hypermetabolism in the cerebellum/dorsal pons, putamen, and primary motor cortex captured the abnormal activity in the cerebello-thalamo-cortical (CbTC) circuits, which was associated with the generation of tremor (Timmermann et al., 2002; Wu et al., 2013). Particularly, the cerebellum, pons, thalamus, and putamen evident overlaps in both CSPTC and CbTC circuits were considered as regions of severer involvement of these circuits. In this study, the dysfunction of CSPTC circuits was involved in functional network abnormalities of PDRP topography. The premotor area receives less excitatory impulses from the thalamus, resulting in a decrease in parietal lobe metabolism (Wu et al., 2013). On the other hand, posterior cortical dysfunction is considered as the imaging marker of PD patients with the risk of dementia (Wu et al., 2013; Peng et al., 2014a). However, understanding the contributions of different brain regions to motor and cognitive impairments requires more relevant cross-sectional and longitudinal studies.

### Structural PDRP Topography

We demonstrated for the first time that spatial covariance analysis can reveal a wide range of regions affected by PD using T1-weighted structural MRI. In this study, a reduced GM volume in PD was observed in bilateral pons, bilateral transverse temporal gyrus, left cuneus, right superior occipital gyrus, and right superior parietal lobule, associated with preservation in GM volume in bilateral pallidum and bilateral putamen. In PD patients, more than 70% of dopamine (DA) terminals were lost when motor symptoms occur (Fearnley and Lees, 1991), and in the early stages of PD, human dyskinesia might be due to the compensatory mechanism that promoted the release and renewal of DA and reduced the uptake of DA so that the DA concentration was stable at normal levels (Silverdale et al., 2003). Besides, there are other possible compensatory changes, including the increase or appearance of striatum TH+ neurons and enhanced DA synthesis by alternative biochemical pathways, etc. These changes might preserve the GM volume in pallidum and putamen in early PD patients (Blesa et al., 2017). Pathological studies related to PD also indicated that the progression of lesions begins with the brainstem (which includes pons) and the substantia nigra (Braak et al., 2003). An decrease in gray volume in the pons has also been reported in previous studies (Jubault et al., 2009). Therefore, the finding in this study is consistent with the pons as the anatomical starting point of PD pathology according to Braak et al. (2003, 2004) and Jubault et al. (2009). In our study, morphological abnormalities in other cortical regions were also found. In cognitively intact PD patients, cortical morphology may be normal (Tessitore et al., 2012) or abnormal in the frontal lobe (Biundo et al., 2011) or in a wider range of cortical regions, including the parietal, temporal, and occipital lobes (Jubault et al., 2011). Uncoordinated results may be caused by different experimental methodologies or the heterogeneity of PD disease.

### Correlations Between PDRP and Clinical Scale

There was no significant correlation between PET-PDRP and MMSE, MRI-PDRP and MMSE, but there were significant positive correlations with UPDRS and six motor items, suggesting that the abnormal metabolic and structural characteristics of nondemented PD patients are related to their dyskinesias, but not to cognitive dysfunction. The injury of PD patients on speech, facial expressions, tremors, rigidity, and gait became worse with the increase in PD pathology, which was consistent with clinical manifestations. Postural tremor is associated with metabolic abnormalities, while stationary tremor is associated with structural abnormalities, indicating that the causes of postural tremor and stationary tremor in PD patients may be different and therapeutic interventions for primary tremor in PD patients need to distinguish postural tremor from resting tremor.

### Correlations Between PET-PDRP and MRI-PDRP

The ROC curves revealed that PET-PDRP and MRI-PDRP expressions significantly discriminated PD patients from the control individuals with approving sensitivity and specificity. Since PET-PDRP expression value was demonstrated reliable diagnosis power, it revealed that MRI-PDRP may be also a promising diagnostic biomarker for the non-inferiority compared to PET-PDRP. However, further validation work should be followed.

As expected, several overlapping regions were identified between the metabolic PET-PDRP topography and the structural MRI-PDRP topography, including the bilateral pons, bilateral pallidum, and bilateral putamen. Surprisingly, the regional weights between the two PDRPs identified in the two imaging modalities were correlated within these regions (*r* = −0.88, *P* = 0.02), and the two pattern expressions were also correlated in patients and normal subjects. In a structural MRI study in combination with deformation-based morphometry and independent component analysis (ICA), researchers identified that the PD-ICA atrophy pattern in a larger number of participants showed a certain spatial topography overlap with the metabolic PD-related pattern derived from spatial covariance analysis using ^18^F-FDG PET (Zeighami et al., 2015). These included the globus pallidus, thalamus, putamen, premotor and supplementary motor regions. Our study obtained a consistent result. The consistent findings indicate a possible link between brain function and structure dysfunction of the related anatomical and functional circuit in PD, particularly the cortico-basal ganglia-thalamocortical motor circuit. In addition, our study demonstrated the potential value of the integration of different neuroimaging techniques to improve the neuropathological understanding of PD. Consistent abnormalities in brain structure and function, and causal relationships between them, in patients with PD await further investigation and understanding.

### Structurally and Functionally Disrupted Network Topology in PD

Brain connectome analysis can reveal abnormalities in network characteristics associated with PD and the potential impact of the disease network on brain information processing. We found that the functional brain network in the two independent PD groups exhibited a disrupted network topology in the disease subspace. Compared with the HC group, the clustering coefficient for the disease subspace was significantly increased, the characteristic path length in the PD1 group was significantly increased, and the small-worldness attribute in the PD2 group was significantly elevated. Similarly, we also found a significantly increased characteristic path length for the brain structure network in PD1 group. An earlier study has used ^18^F-FDG PET data to identify an ROI-based metabolic PD-related pattern, and the disrupted network topology (increased clustering coefficient, reduced characteristic path length, and exaggerated small-worldness attribute) in the disease network consisting of brain regions with significant abnormalities in this pattern has also been confirmed in four independent patient subjects and in an experimental nonhuman primate model (Ko et al., 2017). Consistent with our results, significantly increased clustering coefficients in the disease space could be observed in both studies. However, changes of characteristic path length in the disease space in both studies were against. This may resulted from different disease spaces in morphological topography and nation differences between the western and eastern populations in the two studies. Interestingly, these descriptor changes are limited to the space occupied by the disease network, which might correspond to the relatively intact anatomy of other spaces (nondisease subspace and the whole brain).

## Limitations

There are several issues that still need to be further considered in this study. First, in the pattern analysis, we used the ROI-based rather than the voxel-based SSM/PCA algorithm for the subsequent determination of the disease subspace. The effect of the two different analytical methods on the results needs further investigation. Second, due to limited experimental subjects, for prospective evaluation of PET-PDRP and MRI-PDRP, we only used another new set of PD subjects but did not include both HC and PD subjects. Third, we did not further examine the intrinsic link between pattern and connectome analyses, for example, to see whether the correlation between the metabolic PET-PDRP pattern and structural MRI-PDRP can be further explained from the perspective of connectome analysis.

## Conclusions

In this study, we investigated ^18^F-FDG PET-based metabolic covariance pattern and MRI-based structural covariance pattern associated with PD in the same patients. The metabolic pattern is highly consistent with the disease-related metabolic brain patterns previously described in different cohorts of PD patients. This structural pattern was characterized by relative decreased GM volume in bilateral pons, bilateral transverse temporal gyrus, left cuneus, right superior occipital gyrus, and right superior parietal lobule, associated with preservation in GM volume in bilateral pallidum and bilateral putamen. Expectantly, we found a significantly negative correlation regional weight between metabolic and structural patterns in a set of salient abnormal brain regions, which provides a new perspective for insight into disrupted brain abnormal metabolism and structure in PD patients. In order to verify the effectiveness of two patterns, we used connectome analysis methods to explore the brain metabolic network constructed by PET and the brain structural network. The results showed that more obvious changes could be found in these two patterns. In summary, significant overlaps between metabolic and structural patterns, as well as the convergence of metabolic network and structural network disruption, provide new evidence for elucidating the neuropathological mechanisms of the disease.

## Data Availability Statement

All datasets generated for this study are included in the article/Supplementary Material.

## Ethics Statement

The studies involving human participants were reviewed and approved by Research Ethics Committee of Huashan Hospital, Shanghai, China (approval number: KY2013-336). The patients/participants provided their written informed consent to participate in this study.

## Author Contributions

CL, HZho, and HZha: analysis and interpretation, writing of manuscript. MW and JuJ: study supervision, critical revision of the manuscript for important intellectual content. PW, JG, and JW: acquisition of data, writing of manuscript. JiJ, YM, and CZ: study concept and design, organization, critical revision of the manuscript for important intellectual content.

## Conflict of Interest

The authors declare that the research was conducted in the absence of any commercial or financial relationships that could be construed as a potential conflict of interest.

## Footnotes

^1^http://www.feinsteinneuroscience.org

^2^http://www.brain-connectivity-toolbox.net/
