# Supplementary material for: Brain Functional and Structural Signatures in Parkinson’s Disease
Source: Front Aging Neurosci. 2020 May 26;12:125. doi: 10.3389/fnagi.2020.00125 (PMC7264099; doi:10.3389/fnagi.2020.00125)
Supplement: Supplementary file 1 [file Table_1.DOCX]

**Supplementary material**


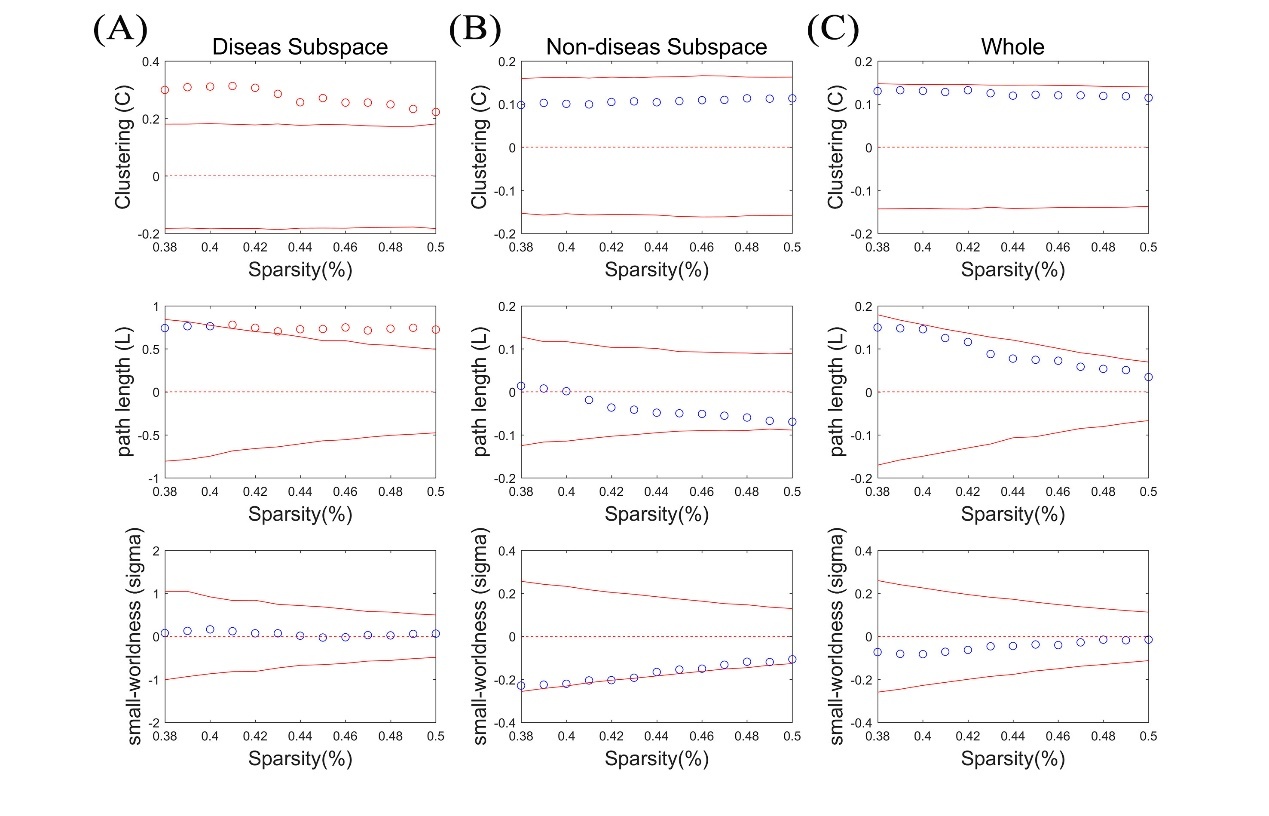


**Figure S1.** Statistical analysis of network metrics between PD1 group and HC group based on ^18^F-FDG PET data. Group differences in the disease subspace (A), non-disease subspace (B), and whole brain (C) for characteristics of clustering coefficient (top), characteristic path length (middle) and small-worldness coefficient (bottom) for PD1 compared with the corresponding HC values (red solid lines = 95% confidence interval, the dotted red line indicates differences equal to 0). The data show that the clustering coefficient for the disease subspace in the PD1 group was significantly (P < 0.05) increased compared with the HC group, and the characteristic path length was significantly (P < 0.05) increased in the same group. However, significant differences were not observed for the small-worldness coefficient. Group differences were also not observed in the non-disease subspace or over the brain as a whole. [Each network metric was computed by varying sparsity from 38-50% over 1000 iterations (see Methods). The red solid lines denote the 95% confidence interval for PD1 vs. HC group differences in each network metric at the corresponding sparsity threshold. Values outside the confidence intervals are significantly different from the permuted group contrasts (P < 0.05, two-tailed, red circles).]


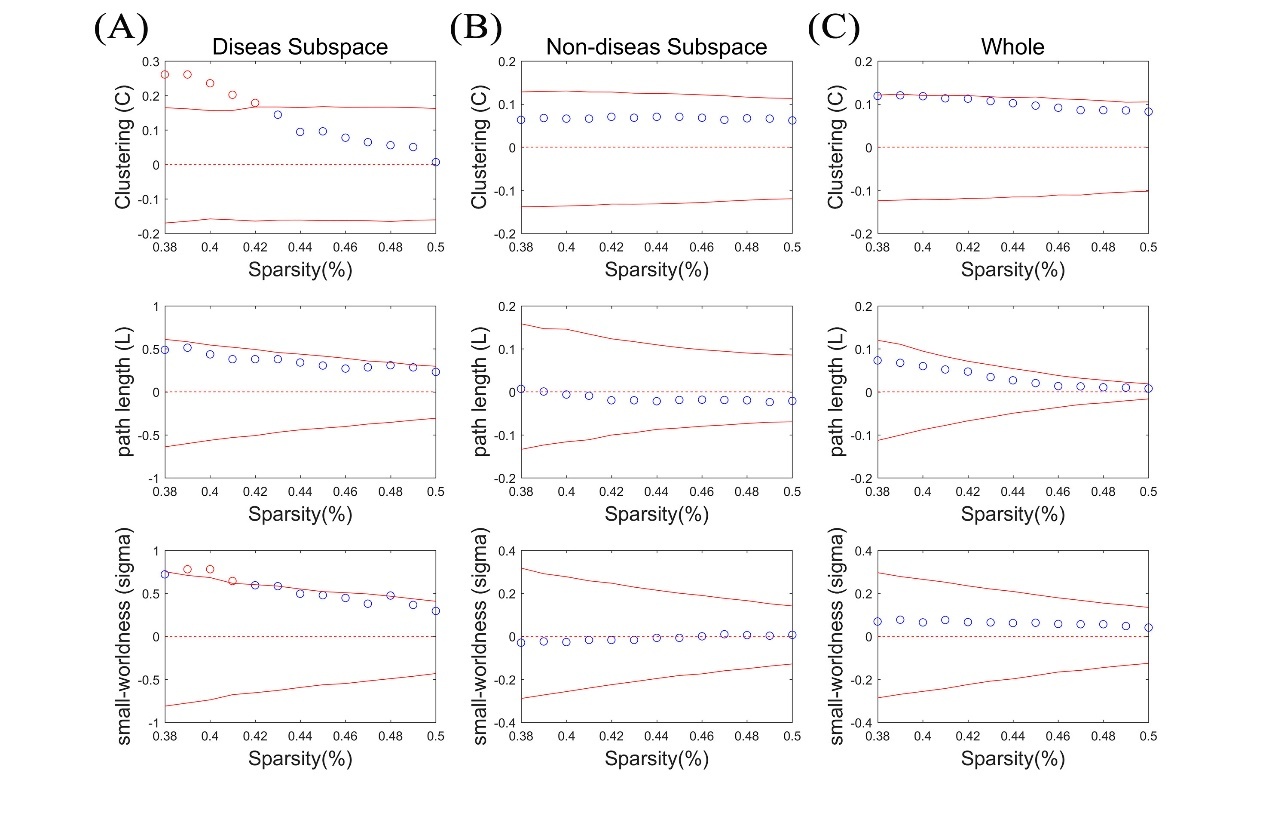


**Figure S2.** Statistical analysis of network metrics between PD2 group and HC group based on ^18^F-FDG PET data. Group differences in the disease subspace (A), non-disease subspace (B), and whole brain (C) for characteristics of clustering coefficient (top), characteristic path length (middle) and small-worldness coefficient (bottom) for PD2 compared with the corresponding HC values. The data show that the clustering coefficient and small-worldness coefficient for the disease subspace were significantly (P < 0.05) increased between patients and controls, but the characteristic path length (had a tendency to increase) showed no group differences. Group differences in network metrics were not significantly different for the non-disease subspace or for the whole brain. The legend description is similar to Figure S1.

**
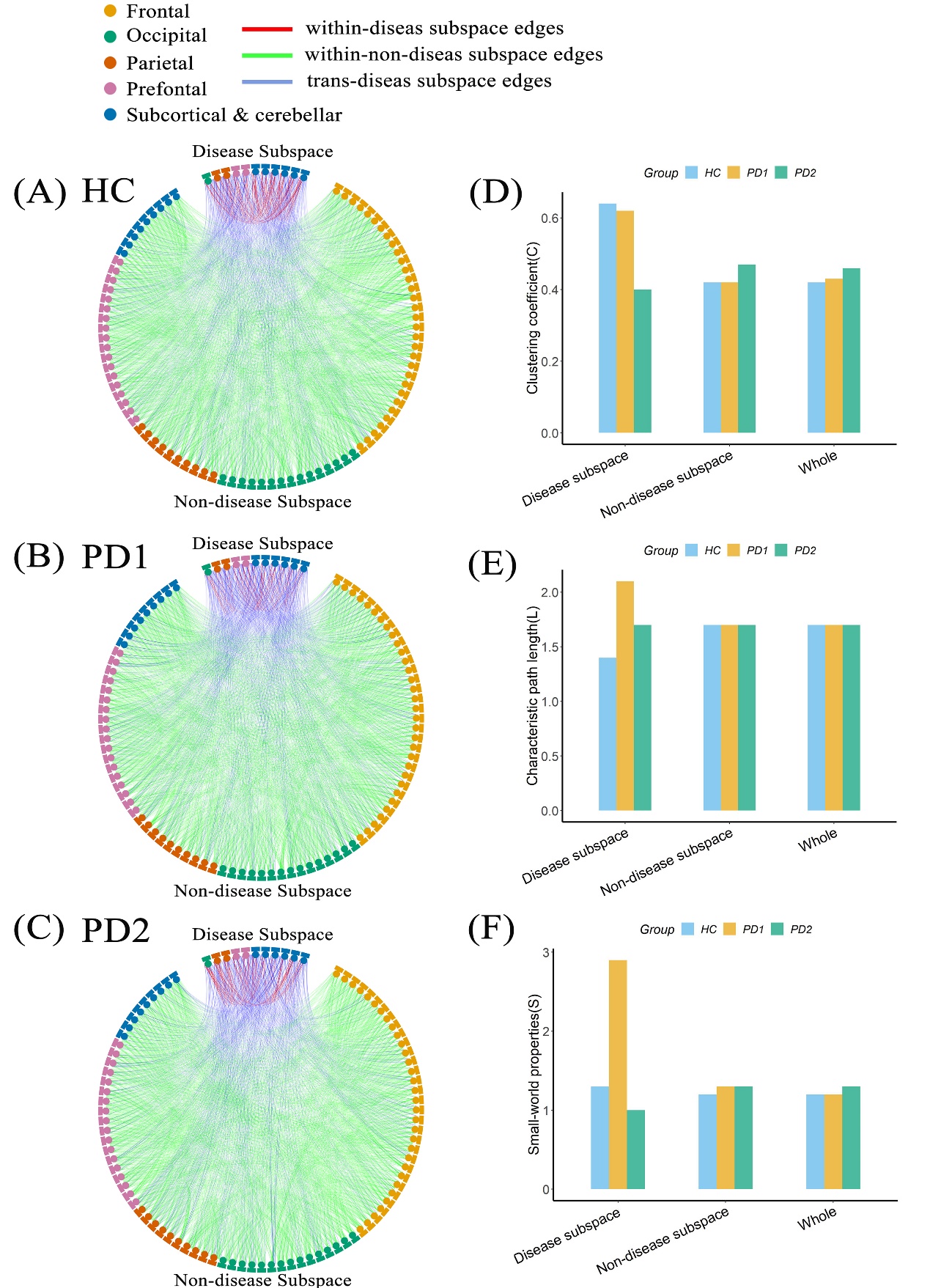
**

**Figure S3.** Node-to-node structural connectivity in the HC (A), PD1 (B) and PD2 (C) groups and network metrics (D, E, and F). (A) Binary structural network in HC group at sparsity 32%. (B) Binary structural network in PD1 group at sparsity 32%.(C) Binary structural network in PD2 group at sparsity 32%. (D) Comparison of the clustering coefficient among HC, PD1 and PD2 groups in different spaces at sparsity 32%. (E) Characteristic path length. (F) Small world coefficient. In (A), (B) and (C), the representation of the legend was the same as in Fig.6.


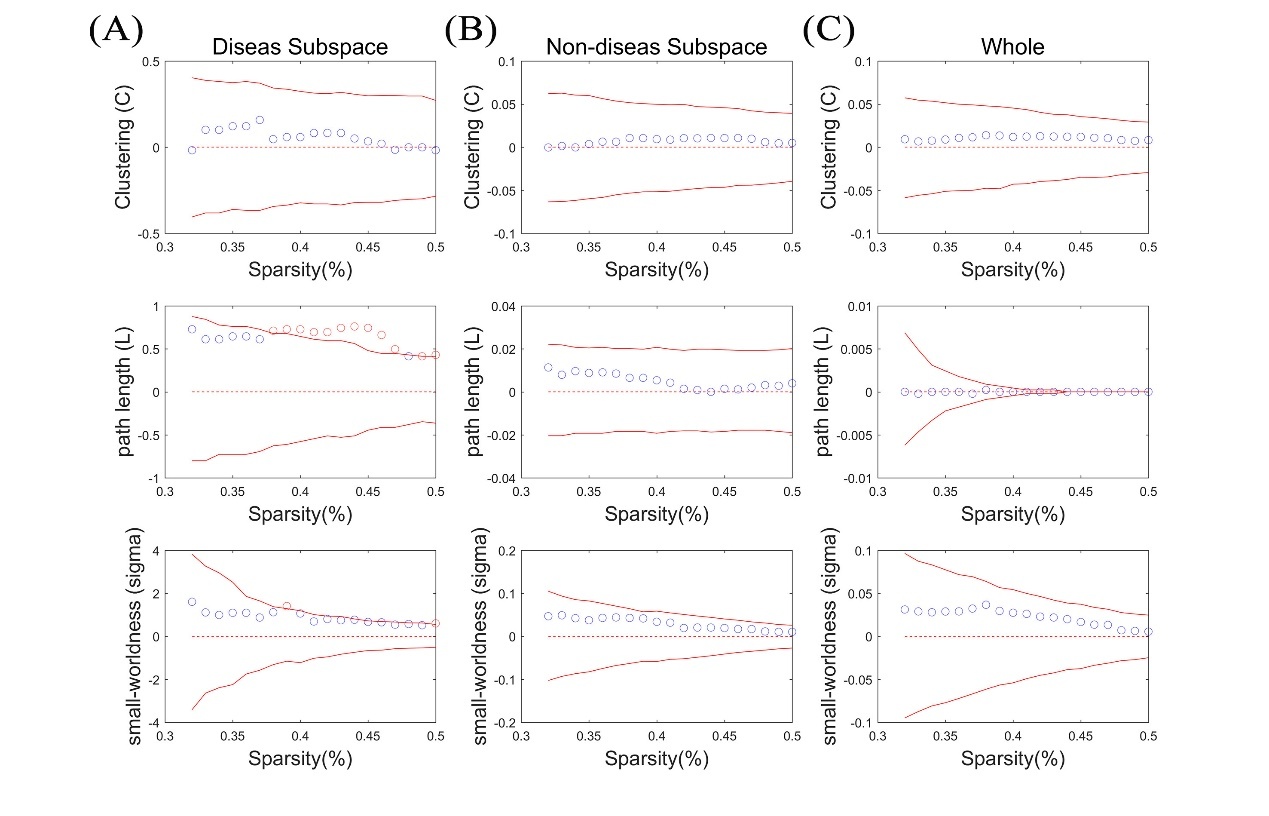


**Figure S4.** Statistical analysis of network metrics between PD1 group and HC group based on T1-weighted GM MRI data. Group differences in the disease subspace (A), non-disease subspace (B), and whole brain (C) for characteristics of clustering coefficient (top), characteristic path length (middle) and small-worldness coefficient (bottom) for PD1 compared with the corresponding HC values. The data show that the characteristic path length and small-worldness coefficient for the disease subspace in the PD1 group was significantly (P < 0.05) increased compared with the HC group, and the clustering coefficient. However, significant differences were not observed for the clustering coefficient. Group differences were also not observed in the non-disease subspace or over the brain as a whole. The legend description is similar to Figure S1.


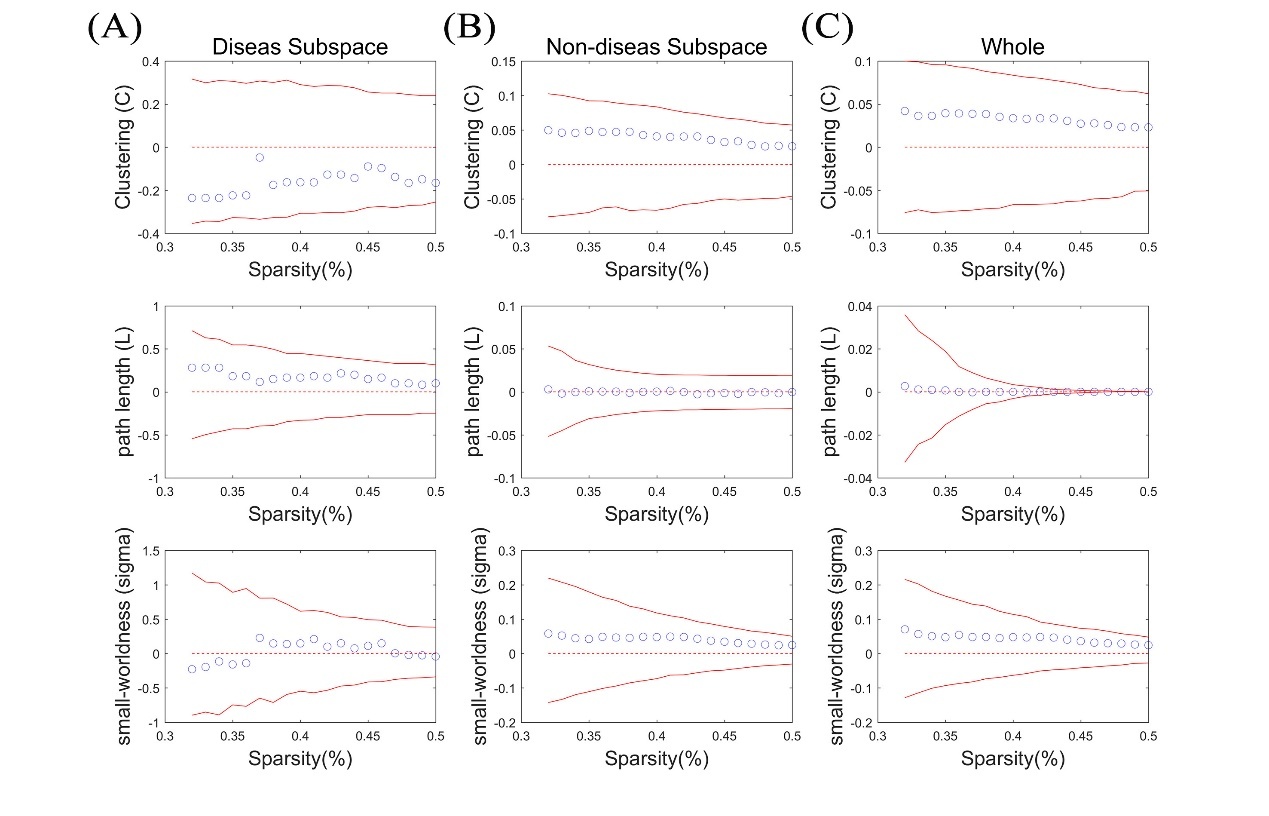


**Figure S5.** Statistical analysis of network metrics between PD2 group and HC group based on T1-weighted GM MRI data. Group differences in the disease subspace (A), non-disease subspace (B), and whole brain (C) for characteristics of clustering coefficient (top), characteristic path length (middle) and small-worldness coefficient (bottom) for PD2 compared with the corresponding HC values. The data show that group differences were not observed in the disease subspace or non-disease subspace or over the brain as a whole. The legend description is similar to Figure S1.

**Table S1** Salient abnormal brain regions (nodes in disease subspace) derived from PET-PDRP topography.

| Index | Region | PET-PDRP  regional weight | MRI-PDRP  regional weight |
| --- | --- | --- | --- |
| 1 | Cingulum_Ant_R | -1.00 | 0.25 |
| 2 | Hippocampus_R | 1.05 | 0.63 |
| 3 | Amygdala_L | 1.13 | 0.26 |
| 4 | Amygdala_R | 1.05 | -0.05 |
| 5 | Occipital_Mid_L | -1.46 | -0.25 |
| 6 | Occipital_Mid_R | -1.60 | -0.18 |
| 7 | Occipital_Inf_L | -1.17 | 0.71 |
| 8 | Parietal_Inf_R | -1.29 | -0.62 |
| 9 | Angular_L | -1.59 | -0.05 |
| 10 | Angular_R | -1.82 | -0.43 |
| 11 | Paracentral_Lobule_L | 1.60 | 0.49 |
| 12 | Paracentral_Lobule_R | 1.94 | -0.34 |
| 13 | Caudate_L | -2.47 | -0.45 |
| 14 | Caudate_R | -2.69 | 1.00 |
| 15 | Putamen_L | 1.31 | 2.08 |
| 16 | Putamen_R | 1.65 | 2.46 |
| 17 | Pallidum_L | 1.10 | 2.64 |
| 18 | Pallidum_R | 1.11 | 4.90 |
| 19 | Thalamus_L | 1.78 | 0.35 |
| 20 | Thalamus_R | 1.45 | 0.70 |
| 21 | Cerebelum_L | 2.21 | -0.04 |
| 22 | Cerebelum_R | 2.34 | -0.30 |
| 23 | Pons_L | 1.86 | -3.49 |
| 24 | Pons_R | 2.14 | -3.15 |
| 25 | Vermis | 2.84 | -0.43 |

**Table S2** Salient abnormal brain regions (nodes in disease subspace) derived from MRI-PDRP topography.

| Index | Region | MRI-PDRP  regional weight | PET-PDRP  regional weight |
| --- | --- | --- | --- |
| 1 | Cuneus_L | -1.15 | -0.23 |
| 2 | Occipital_Sup_R | -1.29 | -0.78 |
| 3 | Parietal_Sup_R | -1.32 | -0.77 |
| 4 | Putamen_L | 2.08 | 1.31 |
| 5 | Putamen_R | 2.46 | 1.65 |
| 6 | Pallidum_L | 2.64 | 1.10 |
| 7 | Pallidum_R | 4.90 | 1.11 |
| 8 | Heschl_L | -1.00 | -0.23 |
| 9 | Heschl_R | -2.28 | -0.03 |
| 10 | Pons_L | -3.49 | 1.86 |
| 11 | Pons_R | -3.15 | 2.14 |
